# Choice of therapeutic strategies in intrathoracic anastomotic leak following esophagectomy

**DOI:** 10.1186/1477-7819-12-402

**Published:** 2014-12-29

**Authors:** Juntang Guo, Xiangyang Chu, Yang Liu, Naikang Zhou, Yongfu Ma, Chaoyang Liang

**Affiliations:** Department of Thoracic Surgery, PLA General Hospital, 28# Fuxing Street, Beijing, China

## Abstract

**Background:**

The aim of this study was to analyze our experience with management of intrathoracic anastomotic leak after esophagectomy.

**Methods:**

Clinical data from 33 patients who developed intrathoracic anastomotic leak were evaluated retrospectively. These patients were selected from 1867 patients undergoing resection carcinoma of the esophagus and reconstruction between January 2003 and December 2012.

**Results:**

Surgical intervention and the reformed “three-tube method” were applied in 13 and 20 patients, respectively. The overall incidence of intrathoracic anastomotic leakage was 1.8%. The median time interval from esophagectomy to diagnosis of leak was 9.7 days. Sixteen patients were confirmed as having leakage by oral contrast computed tomography (CT). Age and interval from surgery to diagnosis of leak were identified as statistically significant parameters between contained and uncontained groups. Moreover, patients with hypoalbuminemia had a longer time to leak closure than patients without hypoalbuminemia. Six patients died from intrathoracic anastomotic leak, with a mortality rate of 18.2%. There was no statistically significant difference in the time to leak closure between patients who underwent surgical exploration and those who received conservative treatment.

**Conclusions:**

Intrathoracic anastomotic leak after esophagectomy was associated with significant mortality. Once intrathoracic anastomotic leakage following esophagectomy was diagnosed or highly suspected, individualized management strategies should be implemented according to the size of the leak, extent of the abscess, and status of the patient. In the majority of patients with anastomotic leak, we preferred the strategy of conservative treatment.

## Background

Intrathoracic esophagogastric anastomotic leak following esophagectomy is the most feared complication of esophageal resection because it is associated with high morbidity and mortality [1, 2]. The presentation of intrathoracic esophageal leak ranges from patients who are asymptomatic to those with circulatory collapse and multiple organ failure. The severity of presentation is largely dependent on the magnitude of the leak and whether the pleural space is contaminated. Because of this broad clinical spectrum, it is difficult to establish a standard strategy for diagnosis and treatment. Some surgeons recommend aggressive surgery, while others prefer conservative approaches [3–5].

The “three-tube method” is the most widely applied conservative treatment for intrathoracic anastomotic leak in Chinese thoracic clinics; this technique includes application of a thoracic closed drainage tube, gastrointestinal decompression tube, and enteral nutrition tube [4]. This method has been used in place of surgery for some uncontained and early leaks in the past several years. However, it is still unclear whether conservative treatment has advantages over surgical exploration.

In the present study, we retrospectively analyzed patients who suffered from intrathoracic anastomotic leaks. The purpose of the study was to review the clinical features, method of diagnosis, and treatment for patients who developed intrathoracic anastomotic leaks after esophagectomy.

## Methods

### Patients and methods

From 1 January 2003 to 31 December 2012, 1,867 patients underwent resection of esophageal carcinoma with intrathoracic esophagogastric anastomosis at the Chinese PLA General Hospital in Beijing, China. Of the 1,867 patients, 1,718 were men (92%) and 149 were women (8%), and the mean age was 64.5 years (range 32–78 years). The stomach was used for reconstruction in 1,842 patients (98.7%), the jejunum was used in 10 patients (0.53%), and the colon was used in the remaining 15 patients (0.8%). All patients underwent intrathoracic anastomosis. Forty-eight patients who underwent cervical anastomosis were excluded from this study.

All patients underwent subtotal esophagectomy through a left-sided approach. In brief, all resections were performed by initial thoracic exploration and dissociation of the tumor through a posterolateral thoracotomy. The stomach was mobilized through diaphragmic incision, and the procedure is similar to that through laparotomy. The right gastroepiploic artery was reserved carefully. A pyloromyotomy was not routinely performed. A resection of the thoracic duct was not routinely performed. Denudation of the lesser curvature was usually performed and a gastric tube was produced in the pleural cavity. After resection of the specimen, an end-to-side anastomosis was constructed between the esophagus and the stomach. The anastomosis was located above or below the aortic arch according to the location of tumor. All anastomoses were delivered by a circular stapler device (21 to 28 mm in diameter). All anastomoses were reinforced with mattress suture or omentum flap.

Patient information that had been entered into an institutional database was reviewed. An anastomotic leak was identified in 33 (1.8%) patients via clinical and radiologic or endoscopic evidence. The medical records of these 33 patients were retrospectively reviewed for age, sex, details of the surgical procedure, pathologic findings, diagnosis and management of the esophageal leak, outcome, and long-term survival.

### Definition and management of anastomotic leaks

**Figure 1:**
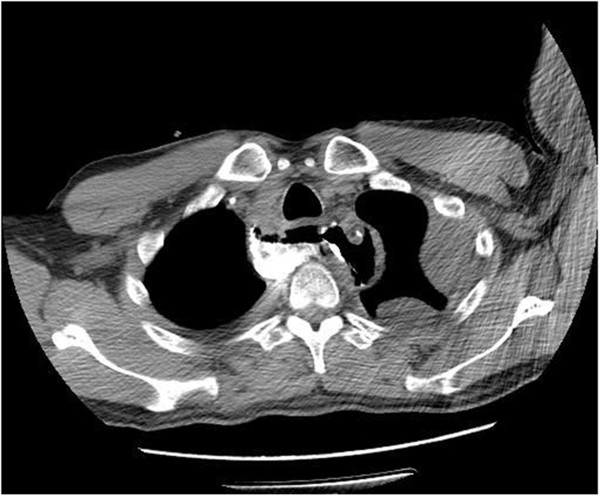
**Oral contrast computed tomography was applied diagnosis of anastomotic leak.** Oral contrast-enhanced transverse computed tomography at day 9. Anastomotic leak with extraluminal contrast and air next to the right wall of anastomosis. An anastomotic leak was viewed at day 12 by endoscopy.

An anastomotic leak was defined as disruption of the esophagogastric anastomosis, the gastric staple line, or both, identified by radiographic contrast examination, operative exploration, or both. The diagnosis of anastomotic leaks was established by three methods: 1) oral methylene blue (n = 12); 2) oral contrast computed tomography (CT; diluted 40 mL compound diatrizoate meglumine or Omnipaque 300; n = 18); and 3) endoscopy or operative exploration (n = 3) (Figure 1) [6, 7]. Anastomotic leak was classified into two types according to the time of development: early leak (within 7 days) and late leak (later than 7 days after operation). According to the severity of the clinical manifestation, anastomotic leak was graded into two types. A contained leak (or minor leak) was defined as a small amount of contrast material around the anastomotic site within the mediastinal space, fever, and an increase in the white blood cell count or C-reactive protein. An uncontained leak (or major leak) was defined as a relatively large amount of contrast material extravasating into the pleural space or draining into the chest tube, the presence of an abscess, mediastinitis, pyothorax, and sepsis [8]. Hypoalbuminemia was diagnosed when the concentration of serum albumin was less than 35 g/L within 1 week after operation. Operative mortality was defined as any death occurring during the first 30 postoperative days or during the same hospitalization period.

Re-operation procedures included anastomosis repair, abscess debridement, thoracic cavity flushing, and placement of several drainage and flush tubes. Conservative treatment choices included restriction of oral intake, administration of intravenous broad-spectrum antibiotics, and application of the reformed “three-tube method”. The three-tube method consisted of placement of a nasogastric decompression tube, a nasojejunum enteral support tube, and a chest drainage tube. Placement of the nasogastric decompression tube and nasojejunum enteral support tube (Freka Endolumina, Fresenius Kabi Company, Bad Homburg, Germany) were guided by endoscopy. A 12-F or 10-F chest tube (SKATER Complete Drainage System, called a pigtail tube; Angiotech Company, Vancouver, Canada) was placed at the bottom of the abscess under the guidance of ultrasonography.

**Figure 2:**
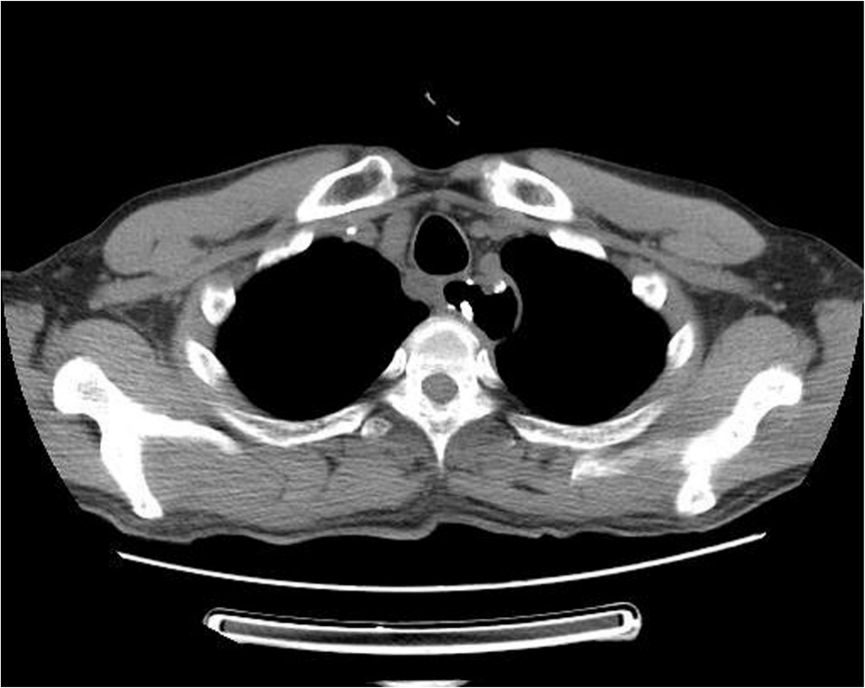
**Oral contrast computed tomography was repeated after 5 weeks.** No extraluminal contrast and air were found. Closure of the anastomotic leak was confirmed by the following oral feed.

Oral contrast CT was carried out after the patient’s temperature and white cell count returned to normal for about 3 to 4 weeks. Oral intake was gradually allowed if the CT showed no contrast medium spilling over into the mediastinum (Figure 2). The time to closure of the leak was defined as the period from the day the leak was diagnosed to the day the leak was confirmed to be closed through follow-up contrast esophagography.

The therapeutic results of the two groups were compared using the χ^2^ test and the *t* test in SPSS version 13.0 software (IBM SPSS, Chicago, USA). Differences were considered significant if two-sided *P*-values were less than 0.05.

## Results

### Patient characteristics and surgery

There were 33 patients (24 men and 9 women) in this study. The median age was 61 years (range 32–74 years). Two (6%) patients with esophageal leaks were asymptomatic. Signs and symptoms were present in the remaining 31 (94%) patients and included fever in 22 patients, shortness of breath in 7 patients, chest pain in 3 patients, atrial fibrillation in 5 patients, pleural effusion in 24 patients, hydropneumothorax in 3 patients, respiratory failure in 4 patients, and renal failure in 1 patient.

Expect for 1 patient with esophageal tuberculosis, 32 patients had malignancies with indications for esophagectomy. The cancer was located at the gastroesophageal junction in 6 patients, medium thoracic esophagus in 21 patients, distal esophagus in 5 patients, and upper thoracic esophagus in 1 patient. The cell type was adenocarcinoma in 5 patients and squamous cell carcinoma in 27 patients. Resection margins were cancer-free in all 32 cases. The cancer was classified as stage I in 5 patients, stage IIA in 10 patients, stage IIB in 3 patients, and stage III in 14 patients. Three patients received neoadjuvant chemoradiation therapy before operation.

Esophagectomy was performed through the left sixth or seventh intercostal space via posterolateral thoracotomy. The stomach was mobilized and was used as a conduit to re-establish gastrointestinal continuity in all patients. Anastomosis was performed via circular staples in all 33 patients.

### Diagnosis and management of leaks

Thirty patients were diagnosed with leaks by assisted examinations that included CT of the chest with oral contrast in 16 (48.5%) patients, oral methylene blue in 10 (30.3%) patients, endoscopic examination in 2 patients, and barium swallow in 2 patients. The accurate rate of CT with oral contrast was 89%. Only 2 patients were not diagnosed with leak by CT with oral contrast and were shown to have leakage by endoscopy. Twenty-five leaks from 31 patients were located in the right wall of the anastomosis. Three patients were diagnosed with anastomotic leaks based on the finding of purulent thoracic effusion or food debris in the incision. The median interval from the initial hypothesis to actual diagnosis of the leak was 4 days (range 0–9 days).

Leaks were limited to the mediastinum, and were therefore considered contained, in 15 patients. Leaks extended into the pleural space in 18 patients and involved the right pleural cavity in 13 patients, the left pleural cavity in 3 patients, and both cavities in 2 patients; all of these were considered uncontained.

The median interval from esophagectomy to diagnosis of the anastomotic leak was 9.7 days (range 1–38 days). There were 16 early leaks and 17 late leaks. Thirteen of the 33 patients underwent surgical exploration, while the other 20 patients were managed conservatively after diagnosis of the leak. In all 33 patients, 28 were fed by enteral nutrition, and 6 were fed by total parenteral nutrition. Among the 18 patients with uncontained leaks, 9 underwent re-operation, and 9 received conservative treatment. Among the 15 patients with contained leaks, 5 underwent re-operation, and 10 received conservative treatment.

### Outcomes

The clinical details of patients with contained and uncontained leaks are compared in Table 1. Age and interval from surgery to diagnosis of leak were significantly different between the two groups. In patients with contained leaks, 14 were cured, while 1 died. In contrast, in patients with uncontained leaks, 12 were cured, while 6 died (1 died of heart failure). There was no significant difference in the cure rate between the two groups.

**Table 1:** **Clinical data of 33 patients with intrathoracic anastomotic leak**

	Group		*P*-value
	Contained leak	Uncontained leak	
Number of patients (n)	15	18	
Age (years)	56.2 ± 11.0	64.8 ± 6.6	0.01* (t-test)
Gender (n)			0.18
Male	10	14	
Female	5	4	
Tumor location (n)			0.6
Upper third	0	1	
Middle third	9	9	
Lower third/GEJ	6	8	
Tumor type (n)			0.01* (χ^2^ test)
SCC	12	15	
ADC	3	2	
Neoadjuvant therapy (n)			0.64
None	14	16	
CT	1	1	
RT	0	1	
TNM stage (n)			0.23
I	4	1	
II	6	7	
III	5	9	
Subgroup (n)			0.77
Early leak	5	11	
Late leak	10	7	
Interval of diagnosis of leak (days)	12.6 ± 8.9	6.9 ± 3.9	0.019* (t-test)
Treatment of leak (n)			
Conservative	11	9	
Surgical exploration	4	9	
Outcome of treatment (n)			0.118
Cured patients	14	12	
Hospital mortality	1	6 (1 died of heart failure)	

*P < 0.05. ADC adenocarcinoma; CT, chemotherapy; GEJ, gastroesophageal junction; RT radiotherapy; SCC squamous cell carcinoma.

Differences in the mean time to leak closure in relation to several variables (age, extent of leak, hypoalbuminemia, and treatment for the leak) are listed in Table 2. The mean time to closure of the leak was significantly longer when the patients had hypoalbuminemia.

**Table 2:** **Time to closure of anastomotic leaks according to the indicated variables in 26 patients (excluding 7 patients with in-hospital mortality)**

Variables	Time to closure	*P*-value
	(days, mean ± SD)	
Age (n)		0.29
≤60 years (14)	44.6 ± 16.0	
>60 years (12)	54.0 ± 27.9	
Extent of leak (*n*)		0.126
Contained (14)	43.7 ± 19.4	
Uncontained (12)	57.5 ± 24.5	
Hypoalbuminemia (n)		0.001* (t-test)
Yes (8)	71.6 ± 21.4	
No (18)	36.9 ± 10.3	
Treatment of leak (n)		0.09
Conservative (16)	41.9 ± 13.4	
Surgical exploration (10)	60.3 ± 29.2	

*P < 0.05; SD, standard deviation.

Seven patients died within 30 days postoperation. Among these seven patients, six succumbed to intrathoracic anastomotic leak. The incidence of operative mortality was 18.2%. Follow-up was complete in all 27 survivors and ranged from 3 months to 10 years (median, 19 months). Sixteen patients required anastomotic dilatation due to anastomotic stricture.

## Discussion and conclusions

Although occurrence of intrathoracic anastomotic leak remains relatively low in China, it is one of the most feared complications of esophageal resection because it is associated with a high mortality rate [9–11]. In our present study, intrathoracic anastomotic leaks occurred in 1.8% of our patients; this was slightly lower than the previously reported incidence [1, 12, 13]. This can be explained by the fact that the study is a retrospective one with its associated limitations, and some cases might be omitted because of the long time span. Another reason could be application of circular staples generally. Some surgeons also prefer to reinforce the anastomosis by using an omentum flap. Several previous studies have indicated that leak incidence is lower with staples than hand-sewn sutures [14–16], although a recent systematic review and meta-analysis revealed no differences when comparing hand-sewn sutures versus staples [17]. However, the use of circular staples increased the risk of developing anastomotic strictures in comparison with using the hand-sewn method, especially in patients treated using staplers with diameter ≤25 mm [17].

The presentation of intrathoracic esophageal leak ranges from patients who are asymptomatic to those with circulatory collapse and multiple organ failure. We agree with the classification of anastomotic leak by Cafarotti [18]. In our present study, age and interval from surgery to diagnosis of leak were identified as statistically significant parameters between patients with contained and uncontained leaks. Late leaks tend to be minor and contained [19, 20].

CT with oral radiographic contrast is a feasible and sensitive way to diagnose anastomotic leak [6, 7, 21]. In our study, nearly half of the patients were diagnosed by CT with oral water-soluble contrast. Chest CT can also allow observation of the magnitude and location of the leak and extent of the abscess, which is helpful for placement of the chest flush and drainage tube. Some investigators have recommended endoscopy for both diagnosis of the leak and assessment of gastric conduit viability [21–24]. It is proved that endoscopy early after esophagectomy is safe and provides accurate and reliable identification of conduit ischemia that can be used to guide the treatment of these patients. However, we reserve the use of endoscopy in patients with late leaks or who are considered stable due to lack of the endoscopic examination at the bedside.

The most effective treatment option for intrathoracic esophagogastric anastomotic leak is controversial, and there is no standardized treatment algorithm. The management of intrathoracic anastomotic leaks should be individualized and guided by the severity of the symptoms, magnitude of the leak, status of the patient, and experience of the surgeon [4]. In this study, no statistical difference in time to closure of leak was noted between patients who were managed conservatively and those who were managed surgically. There was a higher rate of operative mortality in patients who underwent surgical intervention as compared with patients who underwent conservative treatment, although there was no significant difference in the cure rate between the two groups. However, because this study was retrospective, and the number of patients with leaks was small, we cannot exclude the possibility of selection bias.

Some authors have recommended that initial conservative management be used for subclinical leaks, contained leaks, and leaks identified before the patient resumes oral intake [25, 26]. However, for the past 5 years, we have successfully applied conservative treatments in uncontained, major subgroups. The “three-tube method” is the most widely applied method in Chinese thoracic clinics [4, 27–29]. We reformed the traditional “three-tube method” with special tubes and achieved effective outcomes. Most late leaks were limited to the mediastinum, and this location usually did not require flush and drainage tubes. Therefore, in most cases with late and contained leaks, one nutritional tube was sufficient.

Patients with hypoalbuminemia had a longer time to leak closure than patients without hypoalbuminemia, indicating that nutritional support was an important factor in the management of leaks. Enteral feeding is the first choice because of its efficiency and safety [30, 31].

Several studies have reported that self-expanding metallic stents are successful in the treatment of intrathoracic anastomotic leak [32–34]. Stent implantation has advantages of allowing immediate closure of the leak, earlier oral nutrition, and short hospital stay. However, stents may be associated with some complications, such as fatal hemorrhage and food blockage [27, 35–38]. Recently, endoscopic vacuum-assisted closure has been described as a new effective treatment option for intrathoracic leaks and showed higher effectiveness than stent placement [39]. Regardless, we did not apply these two approaches in the current cases.

In summary, we found that intrathoracic anastomotic leak after esophagectomy was associated with significant morbidity and mortality. CT with oral water-soluble contrast was an effective method for the diagnosis of anastomotic leaks. Therefore, we recommend that once intrathoracic anastomotic leak is confirmed or highly suspected, individualized treatment should be given according to the size of the leak, extent of the abscess, and status of the patient. We preferred conservative treatment in most patients.

## Abbreviations

CT: computed tomography.
